# A Young Child With Global Developmental Delay and Epilepsy Secondary to a Novel Variant of KBG Syndrome

**DOI:** 10.7759/cureus.112628

**Published:** 2026-07-13

**Authors:** Hanin S Ibrahim, Raafat H Jadah

**Affiliations:** 1 Pediatric Medicine, Bahrain Defense Force (BDF) Hospital, Riffa, BHR; 2 Pediatric Neurology, Bahrain Defense Force (BDF) Hospital, Riffa, BHR

**Keywords:** epilepsy, genetic, neurology, pediatric, syndrome

## Abstract

KBG syndrome is a rare neurodevelopmental disorder caused by changes in the ANKRD11 gene (ankyrin repeat domain-containing protein 11). It is typically inherited in an autosomal dominant pattern. This syndrome is characterized by a distinct constellation of clinical findings, including short stature, distinctive craniofacial manifestations, skeletal abnormalities, and abnormal patterns of hair growth. One of the most important features for diagnosing KBG syndrome is the large size of the upper central incisors, which often facilitates the diagnosis between the ages of seven and eight years after the emergence of the permanent teeth. Other common comorbidities include intellectual impairment, developmental delays (especially in speech), behavioral and emotional problems, seizure disorder, and hearing loss. While the diagnosis is often based on clinical features, whole exome sequencing (WES) remains the gold standard for identification of the underlying genetic abnormality in the ANKRD11 gene.

This report presents the clinical findings of a young Bahraini male diagnosed with KBG syndrome to highlight the importance of clinical awareness. By sharing his case, we aim to help healthcare providers to recognize these signs and help these children to get the support they deserve as soon as possible.

## Introduction

KBG syndrome is an uncommon autosomal dominant genetic disorder that is often misdiagnosed [1]. The etiology of this syndrome is due to pathogenic variants in the ANKRD11 gene [2]. KBG syndrome produces a unique combination of clinical and congenital findings that can be diagnosed from the initial physical examination, such as short stature and a unique appearance, including a triangular face, synophrys (fused eyebrows), hypertelorism (widely spaced eyes), bushy eyebrows, prominent ears, wide nasal bridge with a bulbous tip, and anteverted nares. These facial features also contain distinguishing dental findings that include a very long philtrum, thin vermilion border of the upper lip, and macrodontia (the syndrome's hallmark feature), which is present in the permanent maxillary centrals, especially the upper central incisors. In addition, skeletal anomalies (brachydactyly, clinodactyly, and scoliosis) as well as a large anterior fontanelle history with delayed closure can also be present. These patients can exhibit a wide range of neuropsychological conditions, including global developmental delay, expressive language impairment, and behavioral issues such as obsessions or anxiety. Additionally, other patients have been documented to suffer from seizure disorders as well as conductive, sensorineural, or mixed hearing loss, and structural brain abnormalities [3].

Currently, the diagnosis depends only on clinical criteria. However, whole exome sequencing (WES) plays an important role in the molecular confirmation of the diagnosis of individuals with mutations in the ANKRD11 gene [4]. Long-term prognosis for KBG syndrome is generally good, as it generally does not lead to life-threatening conditions. Early diagnosis is essential for providing individualized treatment and psychosocial support, which significantly improves the quality of life and long-term outcomes for children with KBG syndrome [5]. 

## Case presentation

The case describes an 11-year-old Bahraini boy born at full-term (38 weeks gestation) via normal vaginal delivery to consanguineous parents. His birth weight was 3.11 kg, with unremarkable antenatal and postnatal complications. His family consists of four siblings with no history of neurodevelopmental delay.

The patient developed a seizure disorder at the age of 18 months, characterized by upward eye rolling, perioral cyanosis, tongue biting, and frothy oral secretions. These episodes involved tonic posturing of the bilateral upper limbs lasting approximately 10 seconds, which occurred multiple times during the day and were followed by post-ictal drowsiness. Furthermore, his early childhood was marked by significant cognitive-motor delays, including rolling over at six months, sitting unsupported at one year, and starting to walk at approximately three years of age; he also had significant speech delays, as he could only say two-word phrases with poor eye contact and impaired social communication.

Neurological assessment showed a normal muscle tone, and full power (5/5) in all extremities with a deep tendon reflex of plus 2 and no cerebellar signs, with an intact cranial nerve examination, and normal and steady gait. The rest of the systems examinations were unremarkable.

Physical examination revealed obvious dysmorphic features highly suggestive of KBG syndrome in the form of craniofacial findings such as a triangular face, with hypertelorism and bushy, fused eyebrows, a bulbous nasal tip with anteverted nares, and a thin vermilion border of the upper lip. Intraoral examination revealed macrodontia of the permanent upper central incisors, which is the syndrome’s hallmark feature. Skeletal assessment noted short stature, scoliosis, and digital anomalies including brachydactyly and clinodactyly with no neurocutaneous skin lesions.

Based on the patient's dysmorphic features, significant neurodevelopmental delay, and associated epilepsy, WES was performed, which confirmed the presence of a heterozygous variant of the ANKRD11 gene, consistent with a diagnosis of autosomal dominant KBG syndrome. In our case, it was confirmed that the patient has the c.3019C>T variant in the ANKRD11 gene, which results in a premature stop codon in exon 10 (of 14) and is listed in the Human Gene Mutation Database (HGMD) as being associated with KBG syndrome [2].

**Figure 1:**
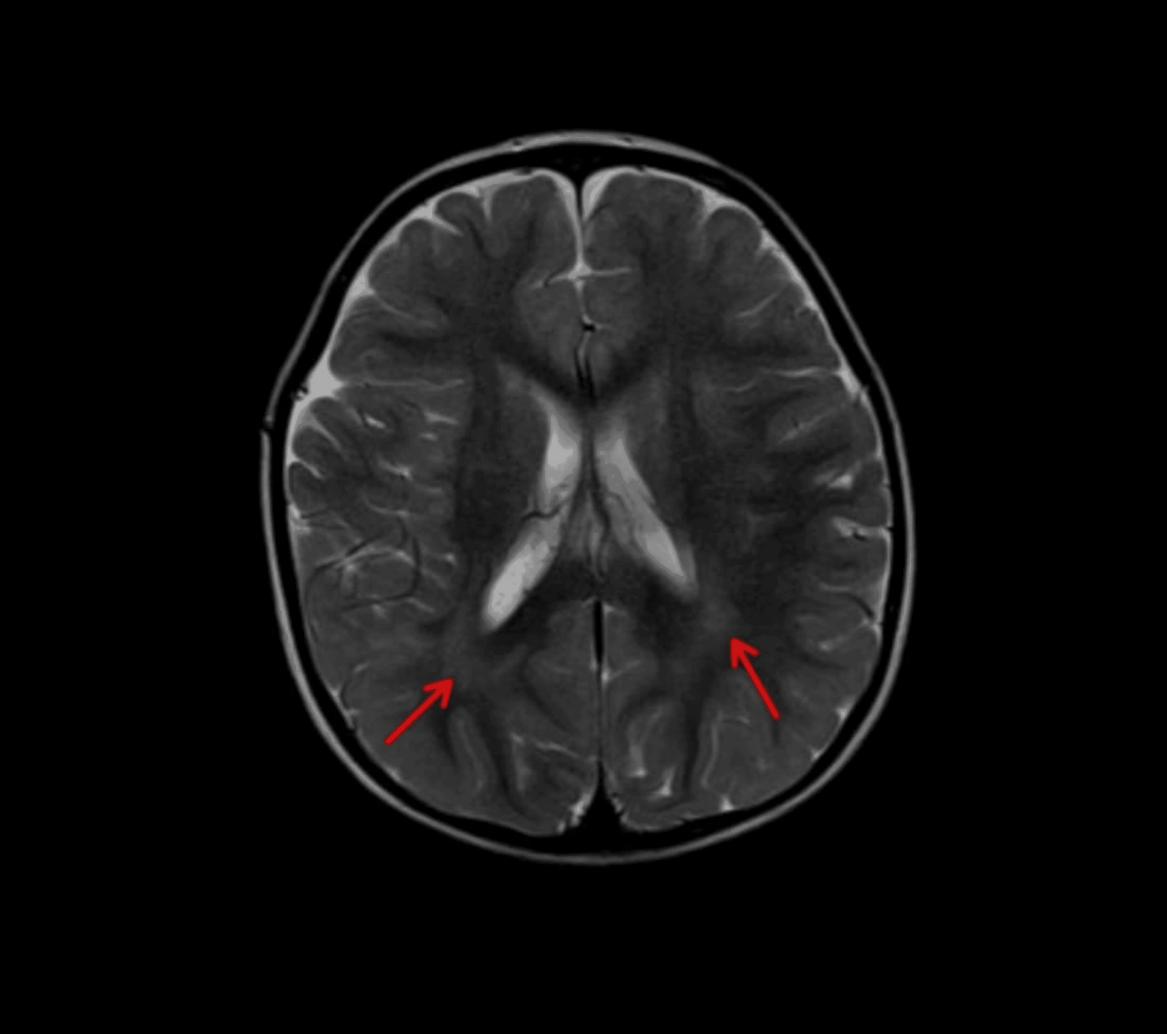
Brain MRI axial view T2 sequence with hypersignal intensities in the occipital head region.

**Figure 2:**
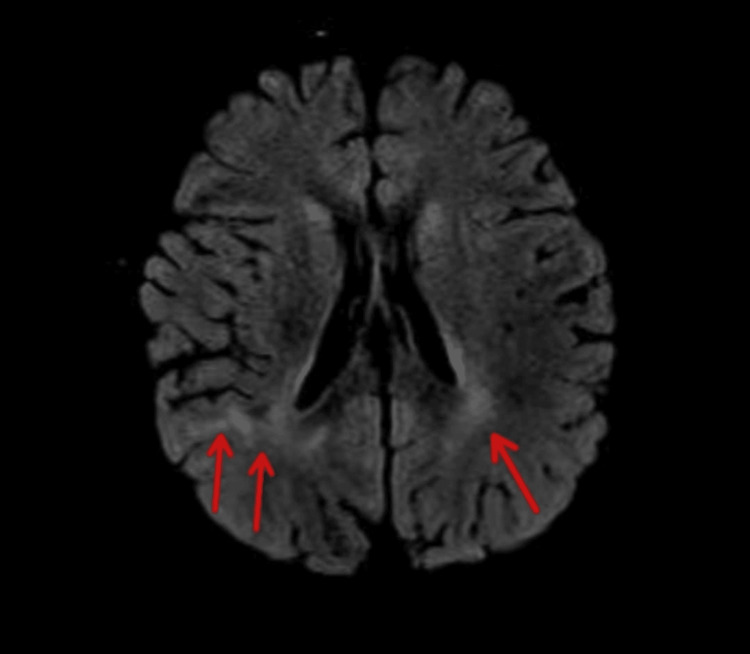
Brain MRI axial view with FLAIR sequence showing multiple posterior hypersignal intensities involving the panventricular, cortical and subcortical areas. FLAIR: fluid-attenuated inversion recovery

While an electroencephalogram (EEG) was normal, brain magnetic resonance imaging (MRI) demonstrated abnormal signal changes in the white matter of the brain, specifically in the occipital area (Figures 1-2).

The patient was initially managed with levetiracetam (Keppra) as monotherapy. However, at the age of 10 years, there was a marked increase in the frequency and severity of seizures. The regimen was optimized by increasing levetiracetam dosage to 60 mg/kg/day and adding lacosamide (Vimpat) at 3 mg/kg/day, which successfully achieved clinical seizure control and better oral and social communication skills with his family.

## Discussion

KBG syndrome is a rare autosomal dominant neurodevelopmental condition that often presents a diagnostic challenge due to its phenotypic heterogeneity [1]. The molecular etiology is primarily attributed to heterozygous pathogenic variants in the ANKRD11 gene. These mutations are localized within exon 9, leading to protein truncation in ANKRD11. Other mutations can be found in patients with c.1903_1907del and c.2398_2401del or deletion in c.2408‐2412del and c.1801C>T, p.(Arg601*), while other cases were reported with microdeletions of chromosome 16q24.3 involving ANKRD11. This gene serves as a critical co-regulator in neurodevelopment and plays an important role in neurogenesis by modulating the proliferation and differentiation of neuronal progenitors [2]. 

The clinical presentation of KBG syndrome is highly variable. One of these variants includes craniofacial dysmorphism with dental anomalies. Most of these cases documented with different degrees of characteristic features including triangular facial shape, synophrys (fused eyebrows), telecanthus, hypertelorism (widely spaced eyes) and associated with other common findings involve a thin vermilion border of the upper lip, a long philtrum, anteverted nares with a broad nasal bridge, and macrodontia of the permanent upper central incisors which remains the clinical hallmark of KBG syndrome. In addition, patients with KBG syndrome can present with a highly variable spectrum of congenital, neurological, and physical anomalies, such as neurodevelopmental delays, which have been reported in 83.3% of pediatric patients with varying degrees of developmental delay, especially in speech, and are associated with behavioral disorders. Psychiatric comorbidities such as depression, hyperactivity, attention deficit, anxiety, aggressive tendencies, and diminished self-confidence are also reported in varying frequencies [3]. 

WES of the ANKRD11 gene has become the gold standard for molecular confirmation, particularly in patients with undiagnosed syndromic features such as KBG syndrome [4]. The prognosis of this syndrome is favorable but remains variable and not associated with serious medical complications. Early diagnosis with intervention will help to provide an effective treatment for patients with KBG syndrome [5]. 

Other variants include skeletal abnormalities, which can occur in 92% of pediatric patients. Radiographic evidence of spinal abnormalities, such as scoliosis, kyphosis, or lordosis, is found in 74% of the cases. The costal and vertebral defects, short stature, and delayed bone age are frequent signs in patients with KBG syndrome. Other skeletal issues include rib anomalies, coccygeal anomalies, brachydactyly, and clinodactyly, which can also be seen in these patients [6]. Furthermore, epilepsy can be found in 50% of patients with KBG syndrome and is frequently associated with EEG abnormalities. Epilepsy onset can occur at any age, extending to adolescence. Currently, most patients demonstrate favorable responses to conventional anti-epileptic drug (AED) therapy, and auditory abnormalities were identified in 25-31% of cases, with conductive hearing loss being the most common finding. The KBG syndrome can present with uncommon features, including genitourinary and cardiac manifestations, which occur in 10-26% of cases. The clinical suspicion for KBG syndrome may be raised in patients presenting with at least two characteristic phenotypic features [7]. 

The management of KBG syndrome is primarily supportive, with a focus on the treatment of underlying complications (e.g., cardiac repair, seizure control, or speech therapy) to optimize the patient’s quality of life [8]. Recent clinical studies have demonstrated that growth hormone (GH) therapy may serve as an effective intervention for managing short stature in affected patients. However, there is no curative treatment for the underlying etiology of KBG syndrome itself [9]. 

## Conclusions

KBG syndrome is a rare hereditary neurological disorder that can be difficult to identify, leaving many families without the answers they need about their child's diagnosis, because this syndrome can manifest itself in different ways. KBG syndrome comprises a variety of congenital and neurological anomalies, including developmental delays. It is very important to have an early diagnosis through genetic testing such as WES and family counseling, so that the child has access to more options for treatment and psychosocial support, which can improve the quality of the child’s life and will help the parents to manage the potential complications associated with the child’s condition. Reporting such a rare syndrome will help both researchers and physicians to develop better diagnostic approaches and provide better care for children with KBG syndrome. 
